# 4355 Impact of Demographic & Racial Differences on DNA Repair Capacity in Lung Cancer

**DOI:** 10.1017/cts.2020.405

**Published:** 2020-07-29

**Authors:** Francesca Christina Duncan, Catherine Sears, Nawar Al Narallah, Ahmad Al-Hader

**Affiliations:** 1 Indiana University School of Medicine

## Abstract

OBJECTIVES/GOALS: Lung cancer is the leading cause of cancer-related mortality in the United States for both men and women. African Americans are disproportionately affected with lung cancer, having higher incidence and mortality rates compared to Caucasian men and women. African American smokers are diagnosed with lung cancer at a lower age with lower cumulative smoking history. Differences in socioeconomic and environmental factors likely contribute to lung cancer disparities, but less is known about acquired biologic alterations that can promote initiation and progression of lung cancer, particularly in African Americans. This is of interest because there may be other biological, genetic, or environmental factors contributing to lung cancer outcomes as it relates to differences in gender and race. One potential biologic variable may be in the DNA repair capacity (DRC), which describes a cell’s ability to repair damage to DNA caused by carcinogens, oxidants, and radiation. Altered DNA repair is a hallmark of cancer, leading to mutations and malignant transformation. We hypothesize that DRC is decreased in African Americans with lung cancer compared to Caucasian Americans with lung cancer, contributing to the disparity that exists in this racial group. We will 1) perform a retrospective chart review to determine demographic differences between African Americans and Caucasians at three central Indiana hospitals and 2) determine the impact of race and lung cancer on DRC amongst African Americans and Caucasians with and without lung cancer. METHODS/STUDY POPULATION: Lung cancer patients are identified in 3 central Indiana hospitals with different payer source and patient populations using ICD codes. Collected demographics include age, gender, pack-years, lung cancer histology, treatment, and mortality. DRC is measured by host-cell reactivation (non-homologous end-joining and nucleotide excision repair pathways) by flow-cytometry. Measurement of DRC is performed on PBMCs obtained from 120 patients (male and female, African Americans and Caucasians with and without lung cancer). Correlation of DRC and lung cancer will be determined by comparing lung cancer diagnosis to quartile DRC, and adjusted for confounders (measured demographics). Correlative measures will include measures of DNA damage and genomic instability. RESULTS/ANTICIPATED RESULTS: 3450 lung cancer patients were diagnosed with lung cancer at Indiana University Hospital between 1/1/2000 – 5/31/2015. Of these, 48.2% were female and 92.7% smokers. African Americans, Caucasians and Other ethnicities represented 12%, 86% and 2%, respectively. Of smokers, 11.4% were African American. The primary payer source was Federal/Medicare. Retrospective review of lung cancer patients from two additional health systems (county and VA hospitals) will be performed as above with outcomes measured. DRC and additional correlative measures will be performed as in Methods. DISCUSSION/SIGNIFICANCE OF IMPACT: If present, altered DRC in African Americans compared to Caucasians may contribute to the disproportional impact of lung cancer on African Americans. If DRC is decreased in African Americans with lung cancer, future studies will focus on identifying potential genetic, epigenetic and environmental causes for this decrease.

